# Supplementary material for: CEP55 as a Promising Immune Intervention Marker to Regulate Tumor Progression: A Pan-Cancer Analysis with Experimental Verification
Source: Cells. 2023 Oct 15;12(20):2457. doi: 10.3390/cells12202457 (PMC10605621; doi:10.3390/cells12202457)
Supplement: Supplementary file 1 [file cells-12-02457-s001.zip › Supplementary figures.pdf]

Supplementary figure legend

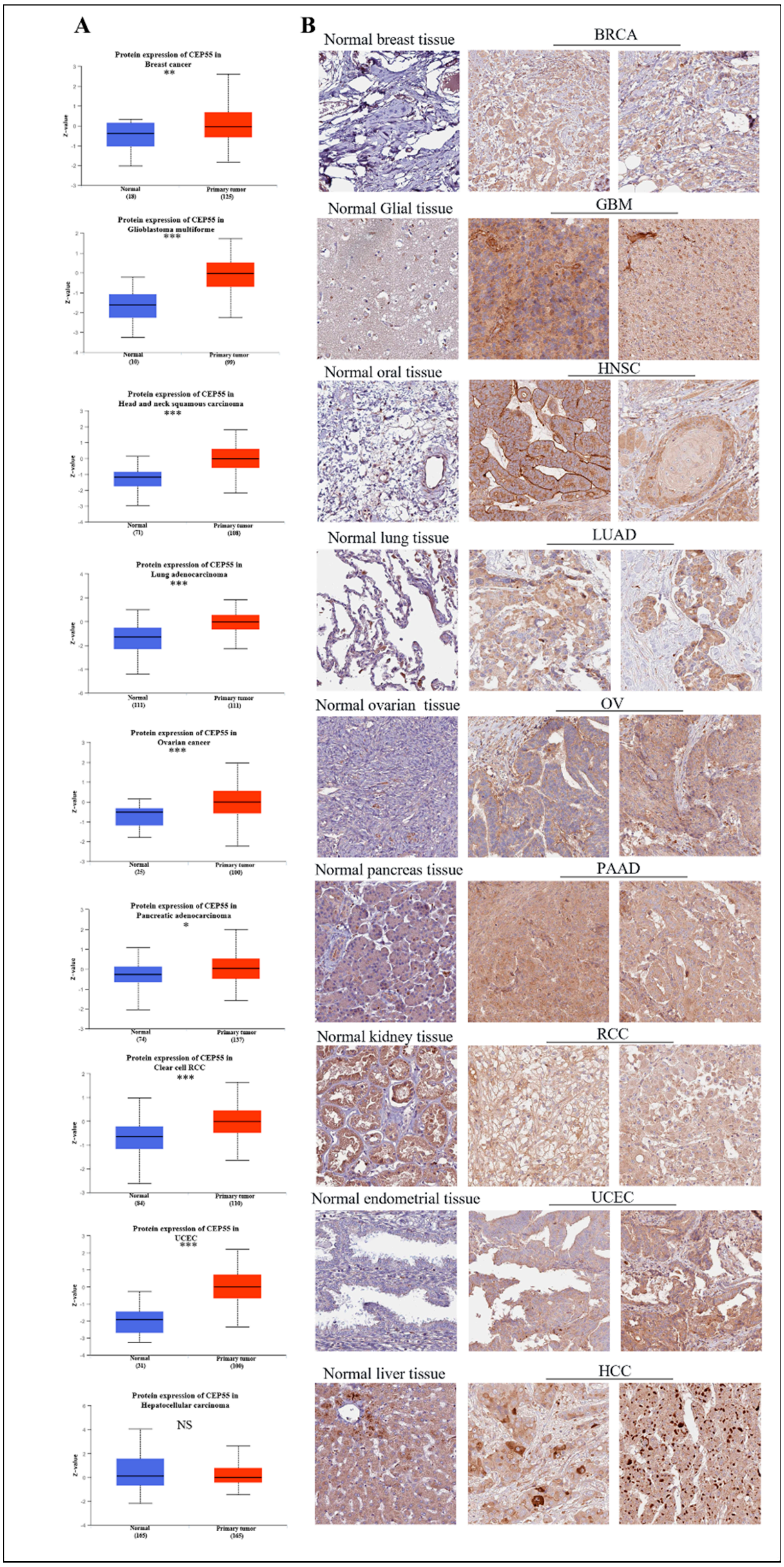

Supplementary Figure S1. Protein expression levels of CEP55 in pan-cancer. (A) Differential levels of CEP55 protein expression in primary carcinomas BRCA, GBM, HNSC, LUDA, OV, PAAD, RCC, UCEC,

HCC and corresponding normal tissues were compared on UALCAN. *P<0.05, **P<0.01, ***P<0.001. (B) The protein expression of CEP55 was compared in immunohistochemical images of normal tissue (left) and tumor tissue (middle and right) on HAP database.

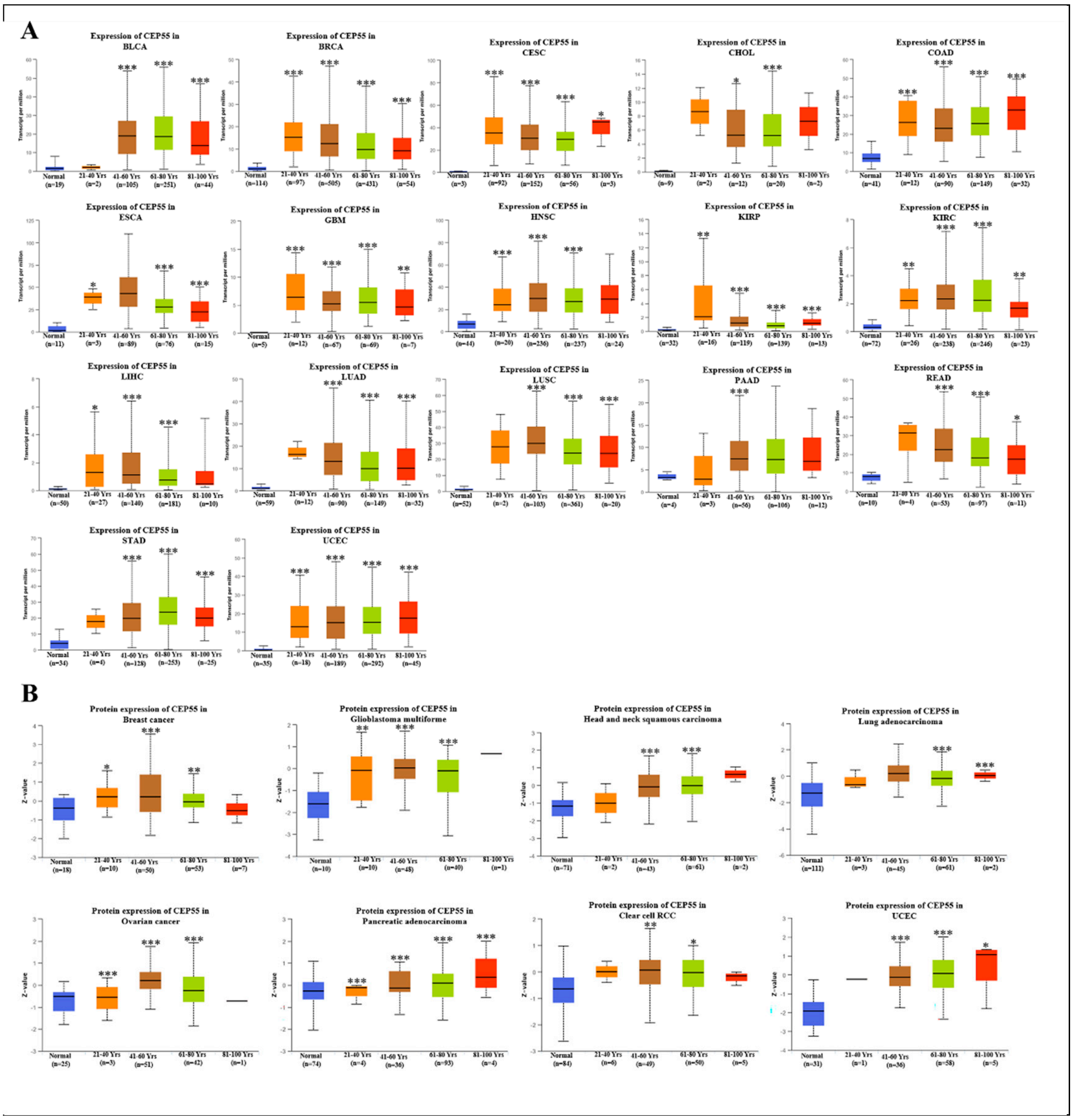

Supplementary Figure S2. Association of CEP55 (A) mRNA expression with age in BLCA, BRCA, CESC, CHOL, COAD, ESCA, GBM, HNSC, KIRP, KIRC, LGG, LIHC, LUAD, LUSC, PAAD, READ, STAD and UCEC; (B) protein levels with age in BRCA, GBM, HNSC, LUAD, OV, PAAD, RCC and UCEC. All data was taken from the UALCAN database. *P<0.05, **P<0.01, ***P<0.001 vs. normal.

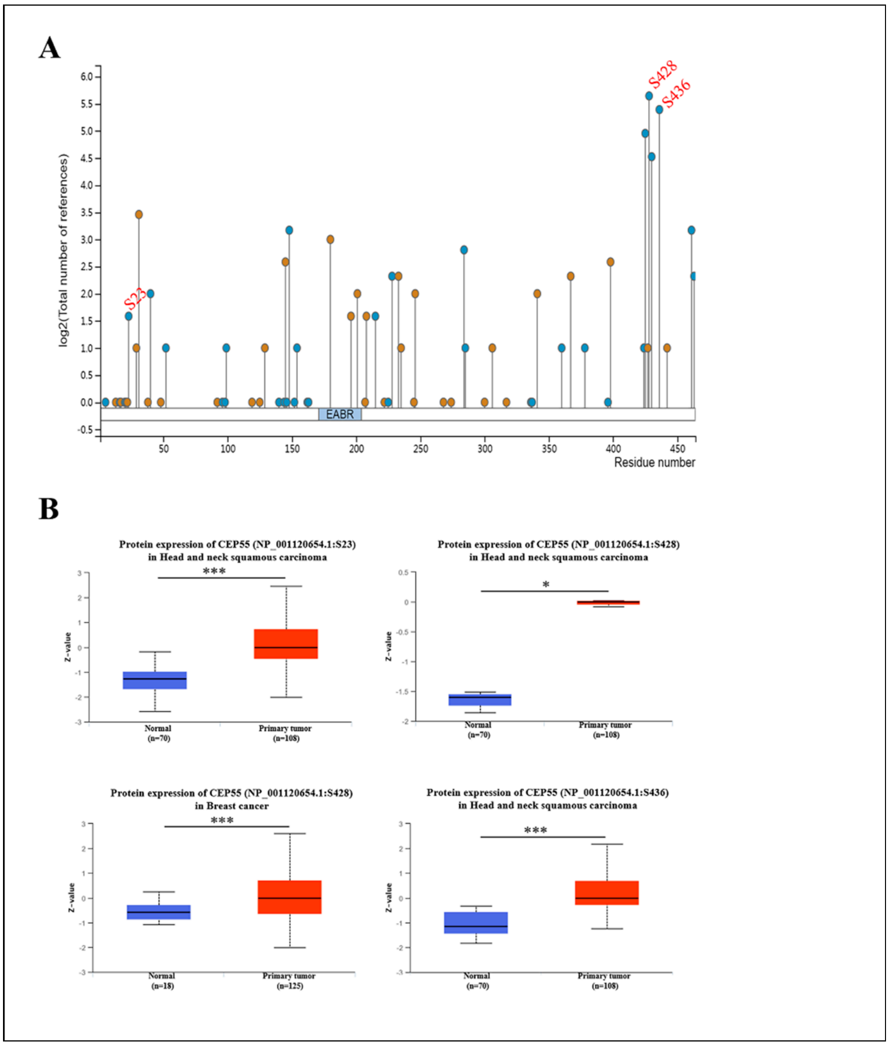

Supplementary Figure S3. Phosphorylation analysis of CEP55. (A) An overview of the phosphorylation sites of CEP55. (B) Phosphorylation levels of CEP55 at different sites in HNSC and BRCA. The results were obtained from the UALCAN database. *P<0.05, **P<0.01, ***P<0.001 vs. normal.

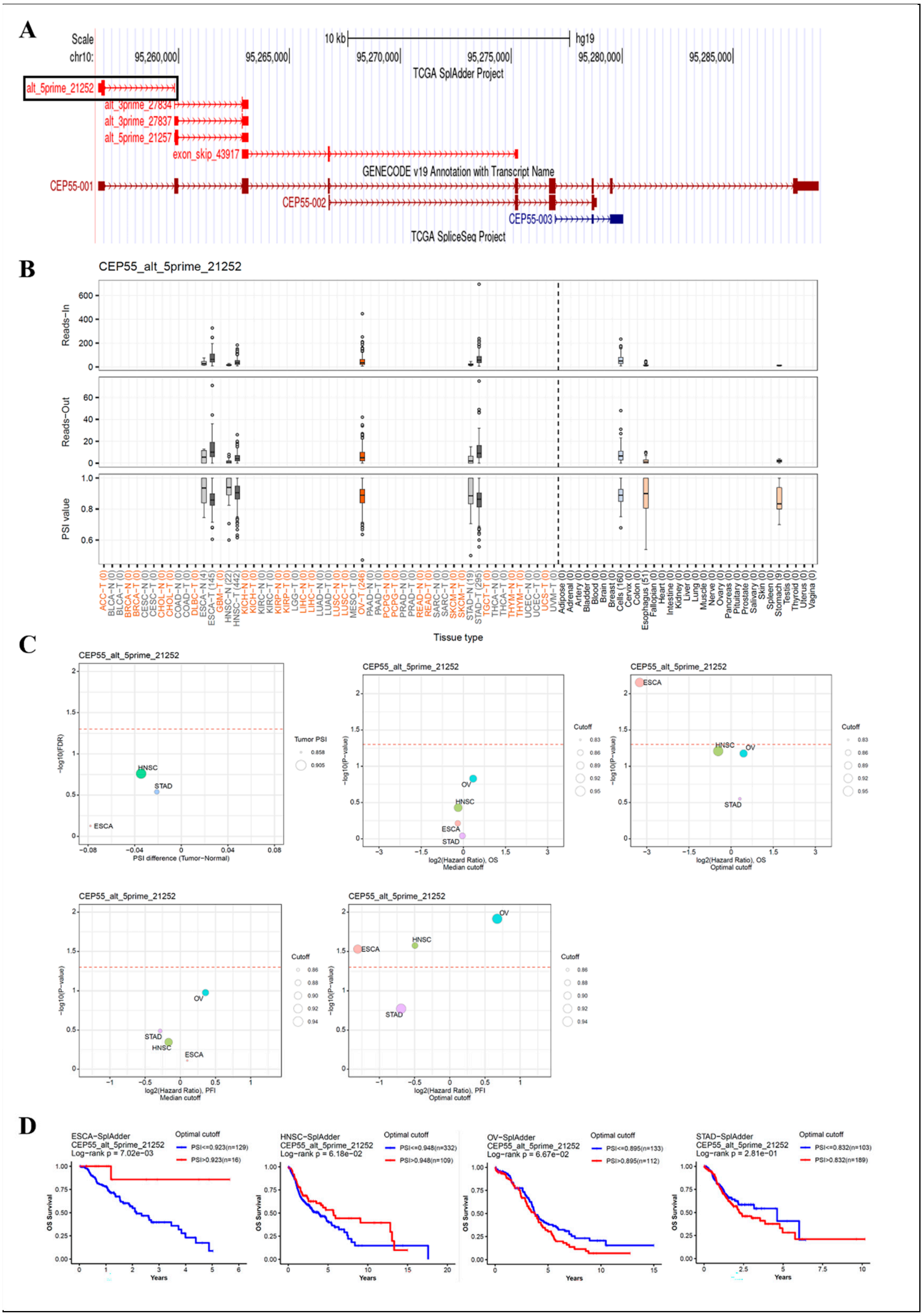

Supplementary Figure S4. Effect of CEP55 alternative splicing on the prognosis of cancer patients. (A) Schematic diagram of CEP55 alternative splicing alt_5primer_51252. (B) The reads-in, reads-out, and PSI value of CEP55_alt_5primer_51252 were analyzed in pan-cancer, adjacent, and normal tissues, respectively. (C) Differences in PSI between tumor, adjacent normal tissue (left) and tumor, GTEx normal tissue (right) and associations with OS and PFI; red dashed line is FDR of 0.05, dot size represents tumor

PSI values, and different colors mark different cancers. (D) Kaplan-Meier curves for patient OS prediction are shown. All data were obtained from OncoSplicing.

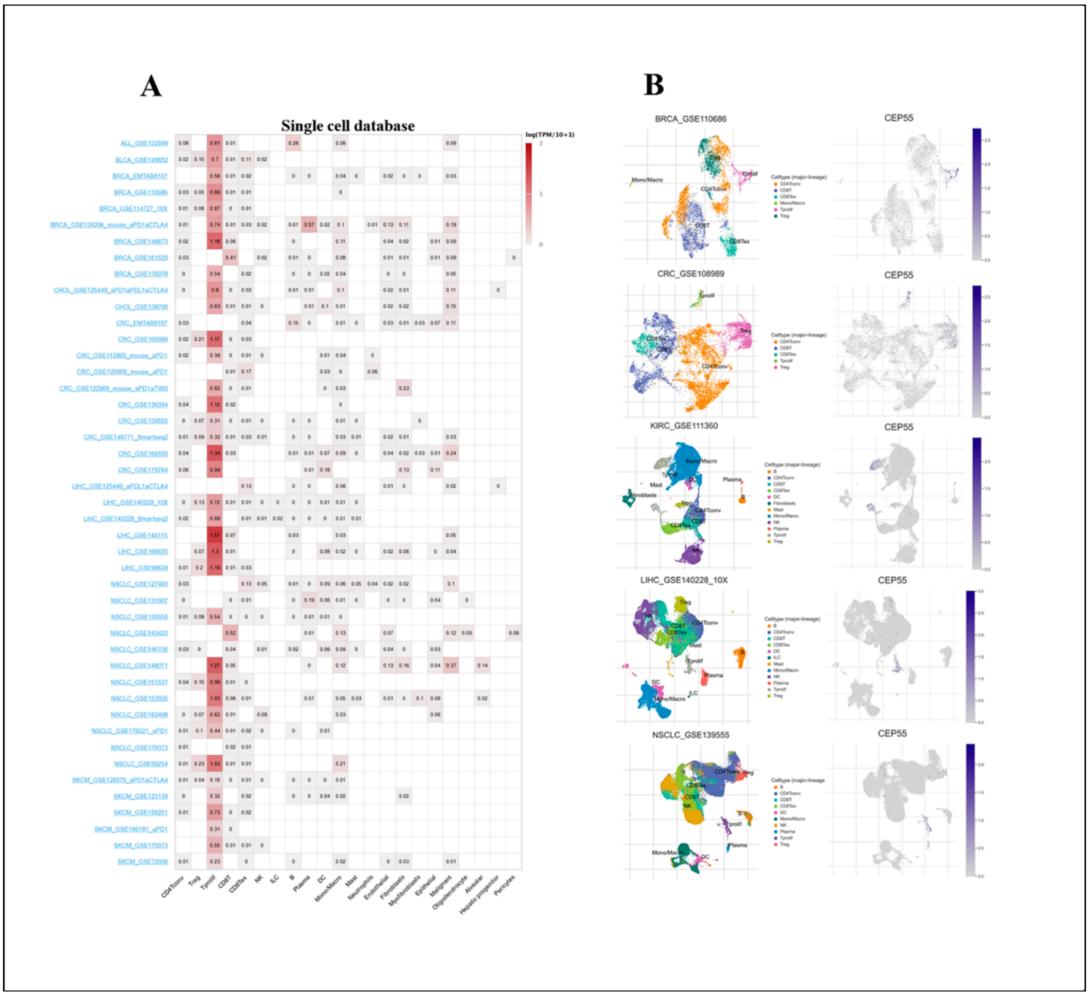

Supplementary Figure S5. Expression analysis of CEP55 in immune cells. (A) CEP55 expression in multiple tumor monocyte clusters. (B) Expression of CEP55 in BRCA, CRC, KIRC, LIHC and NSCLC immune cells. All data were obtained from the TISCH online tool.

Supplementary tables

Supplementary table 1: Tumor samples and classification in TCGA
